# Supplementary material for: Using data derived from cellular phone locations to estimate visitation to natural areas: An application to water recreation in New England, USA
Source: PLoS One. 2020 Apr 30;15(4):e0231863. doi: 10.1371/journal.pone.0231863 (PMC7192446; doi:10.1371/journal.pone.0231863)
Supplement: S1 File — (DOCX) [file pone.0231863.s001.docx]

**Supplementary Materials**

**Title:** Using Datasets Derived from Cellular Phone Locations to Estimate Visitation to Natural Areas: An Application to Water Recreation in New England, USA.

**Authors:** Nathaniel H. Merrill^1^*, Sarina F. Atkinson^2^, Kate K. Mulvaney^1^, Marisa J. Mazzotta^1^, Justin Bousquin^3^

^1^ U.S. Environmental Protection Agency, Office of Research and Development, National Health and Environmental Effects Research Laboratory, Atlantic Ecology Division, Narragansett, Rhode Island, USA

^2^ University of Miami, Miami, Florida, USA

^3^ U.S. Environmental Protection Agency, Office of Research and Development, National Health and Environmental Effects Research Laboratory, Gulf Ecology Division, Gulf Breeze, Florida, USA

* Corresponding author, U.S. EPA, Office of Research and Development, National Health and Environmental Effects Laboratory, Atlantic Ecology Division, 27 Tarzwell Drive, Narragansett, RI 02882, [merrill.nathaniel@epa.gov](mailto:merrill.nathaniel@epa.gov)

**Cell Data Sample Details:**

*Geography*

The set of water access areas, points of interest (POIs), we used consisted of a comprehensive list of all the public beaches and public access points to water (beaches, parks, ways to water, boat ramps) for Barnstable County, Cape Cod, MA compiled from federal, state, county, and town GIS information. The geographic dataset also contains 113 other beaches in greater New England with varying levels of water quality based on bacterial swim advisory or closure history in the last five years. To ensure a range of beaches with different quality were selected, we used the bacterial closure history as a proxy for other parameters because it is consistent and readily available. These additional beaches were randomly chosen across a distribution of beaches within the Beach Advisory and Closing Online Notification (BEACON) dataset^1^.

In consultation with Airsage, we spatially buffered (added area) around the POIs which were designated as line or point features in the spatial database. After attempting a range of spatial buffers, a 100-meter buffer was chosen to balance specificity in capturing water recreation activities (i.e. not capturing ancillary points of interest in geographies, like restaurants or stores for example) with the accuracy of the locational information in the data that Airsage processes. The correct spatial designation and buffers for various applications is certainly an area that future research could address when combined with observations, but is outside of the scope of this paper. The combined and buffered spatial dataset used in this paper is included with the data package.

*Time*

We requested a sample of four months (June, July, August, September) of 2017 at each POI to overlap with the bathing season and our visitation observations. Airsage’s Target Location Analytics (TLA) product provides estimates of unique visitors at locations by hours, groups of hours, and for the full 24 hours of a day. To match these data observations, which were representative of the hours of 9AM-4PM with some small variations, we used the hourly information from Airsage and made assumption on the duration of stay to estimate unique users in the longer 9AM-4PM time window (a specific group of hours not reported by Airsage in terms of unique visitors). The underlying issue is that each hour’s data from Airsage represents unique people in a time window, but someone can stay for multiple hours, and thus be counted twice if we simply sum across hours. In consultation with Airsage, we did the following to their standard TLA product for each POI:

1. Calculated a moving average (3-hour window) of visitation for each hourly visitation estimate.
2. Summed the moving average of the central hour of three-hour blocks from 8AM-4PM (9AM, 12PM, 3PM). This inherently assumes a 3-hour average duration of stay.

By doing this, we created an estimate of visitation from 9AM-4PM from Airsage’s standard TLA product. The 3-hour duration of stay assumption is very general, so we sought to correct any inaccuracies induced by using that assumption by using the visitation model fit to observations in this paper.

**Narragansett Beach Observation Details:**

Because Narragansett Town Beach only collects fees from those that do not have resident passes, their counts only capture a subset of the people that visit the beach. Therefore, we used the relative number of cars between the resident and public parking lots to correct the counts to represent total visitation. We counted on three different days across popular hours. We applied the average ratio between car counts, .85/1, to the counts of public use to create resident totals, which we added to public use for an overall visitation total.

Supplementary Table S1. Performance statistics for candidate models

| Date | Time count was taken | Public | Resident | Ratio (Resident/Public) |
| --- | --- | --- | --- | --- |
| 7/13/2018 | 2-3pm | 251 | 344 | 1.37 |
| 7/24/2018 | 12-1pm | 186 | 125 | 0.67 |
| 7/26/2018 | 3-4pm | 116 | 59 | 0.51 |
|  |  |  | Average: | 0.85 |

*Note:* Car counts of resident and public parking lots were used to find the ratio of resident and public use of the beach. This ratio was applied to daily counts from ticket receipts (public only) to estimate total daily visitation.

**Model Details:**

Data and model code can be found at <https://github.com/USEPA/Recreation_Benefits.git>.

Supplementary Table S2. Candidate Regressions

|  | | | | |
| --- | --- | --- | --- | --- |
|  |  | | | |
|  |  | | | |
|  | Visits | Log(Visits) | Visits | Log(Visits) |
|  | (1) | (2) | (3) | (4) |
|  | | | | |
| Cell data | 0.245^***^ | 0.0003^***^ | 0.296^***^ | 0.0002^***^ |
|  | (0.005) | (0.0000) | (0.007) | (0.0000) |
|  |  |  |  |  |
| Area (m^2^) |  |  | 0.00003 | 0.000006^***^ |
|  |  |  | (0.0002) | (0.0000006) |
|  |  |  |  |  |
| Narragansett |  |  | -646.796^***^ | 0.182 |
|  |  |  | (72.136) | (0.225) |
|  |  |  |  |  |
| Town of Barnstable |  |  | -60.612 | -0.409^***^ |
|  |  |  | (42.119) | (0.131) |
|  |  |  |  |  |
| Temperature (°F) |  |  | 10.398^***^ | 0.066^***^ |
|  |  |  | (2.567) | (0.008) |
|  |  |  |  |  |
| Precipitation (inches) |  |  | -26.180 | -0.447^***^ |
|  |  |  | (47.250) | (0.147) |
|  |  |  |  |  |
| Constant | 33.128^*^ | 4.320^***^ | -334.114 | 0.539 |
|  | (19.496) | (0.064) | (206.779) | (0.644) |
|  |  |  |  |  |
|  | | | | |
| Observations | 352 | 352 | 392 | 392 |
| R^2^ | 0.885 | 0.579 | 0.925 | 0.740 |
| Adjusted R^2^ | 0.885 | 0.578 | 0.922 | 0.729 |
| Residual Std. Error | 319.105 | 1.046 | 268.806 | 0.837 |
| F Statistic | 2,704.218^***^  (df = 1; 350) | 481.175^***^  (df = 1; 350) | 310.289^***^  (df = 15; 376) | 71.207^***^  (df = 15; 376) |
|  | | | | |
|  | ^*^ p<0.10^**^ p<0.05p^***^p<0.01 | | | |

Note: Dummy variables are included for month and day of the week in each regression. Columns 2 and 4 are in log-linear form. See code for additional details and candidate models.

**Random Forest Model Results / Calibration**

Fit using the ranger package in R:

Marvin N. Wright, Andreas Ziegler (2017). ranger: A Fast Implementation of Random Forests for High Dimensional Data in C++ and R. Journal of Statistical Software, 77(1), 1-17.<[doi:10.18637/jss.v077.i01](https://doi.org/10.18637/jss.v077.i01)>

Type: Regression

Number of trees: 2000

Sample size: 392

Number of independent variables: 16

Mtry: 10

Target node size: 5

Splitrule: variance

OOB prediction error (MSE): 70781.18

R squared (OOB): 0.9238585


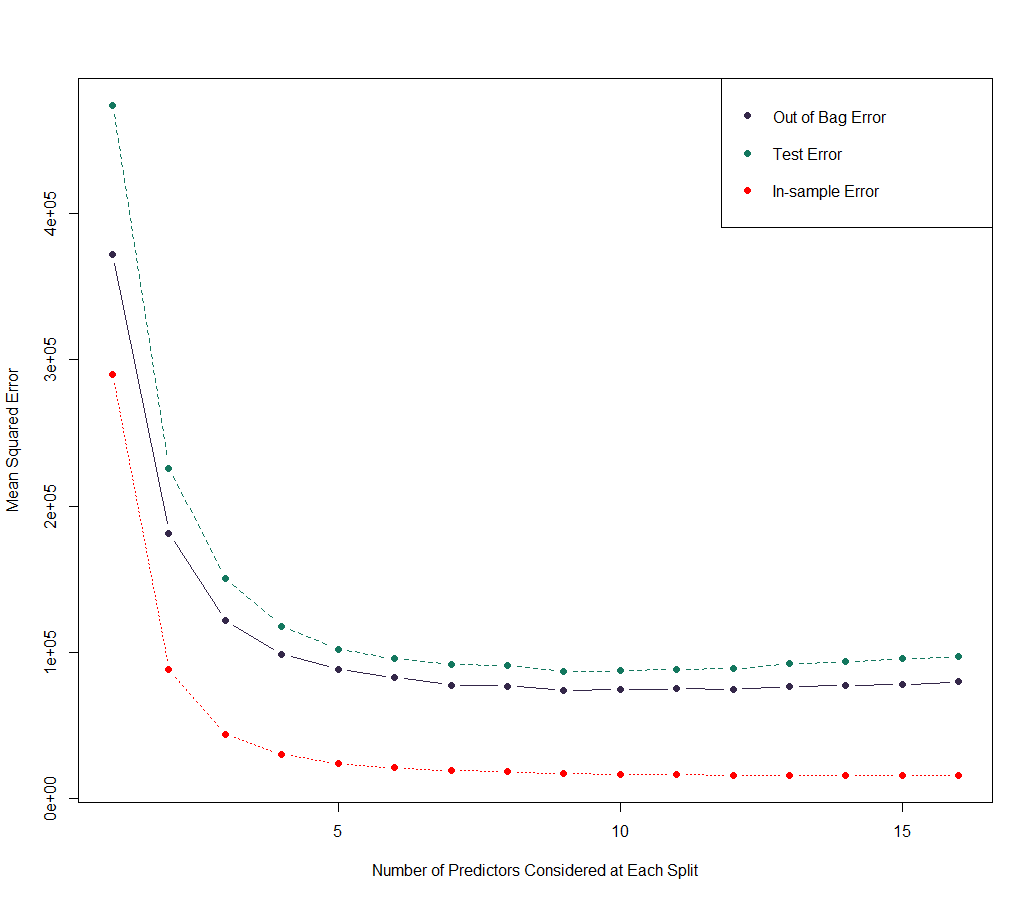


Supplementary Figure S1. Mean squared errors when considering a different number of candidate prediction variables at each split in a tree.


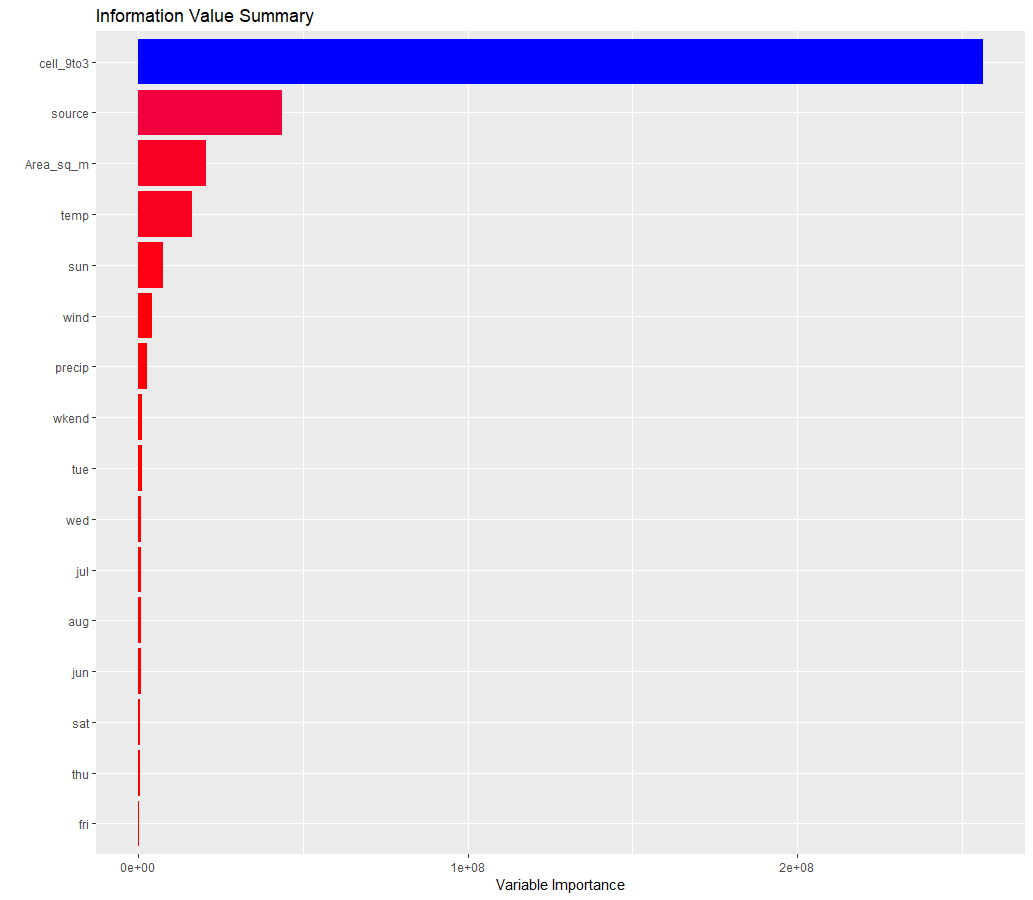


Supplementary Figure S2. Variable importance plot using the ‘impurity’ method in R package Ranger.

**Model Performance Tables**

Supplementary Table S3. Out-of-sample performance statistics for each candidate model

| Candidate Models | Source and Area | Month/ Day | Weather | ME | RMSE | MAE | R-Squared |
| --- | --- | --- | --- | --- | --- | --- | --- |
| Linear models |  |  |  | 0.27 | 318.08 | 186.69 | 0.86 |
|  | x |  |  | 0.21 | 279.61 | 173.11 | 0.88 |
|  | x | x |  | 0.73 | 278.20 | 173.11 | 0.89 |
|  | x | x | x | 0.43 | 272.47 | 174.68 | 0.89 |
| Log-linear models |  |  |  | -430.96 | 3161.23 | 791.33 |  |
|  | x |  |  | -96.47 | 1348.39 | 415.38 |  |
|  | x | x |  | -117.09 | 1399.99 | 441.11 |  |
|  | x | x | x | -74.35 | 1030.21 | 345.93 |  |
| Random forest | x | x | x | -3.78 | 262.48 | 154.84 | 0.91 |

*Note:* Each candidate model is presented with varying explanatory variables and functional form. ME= mean error, RMSE = root mean squared error, MAE = mean absolute error, MAPE = mean absolute percent error, R-squared= % of Out-of-Sample Variance Explained.

**Regression on Each Visitation Data Source**

Since our observational data came from three sources using variations on how the visitation records were compiled, as a check, we ran the fully specified linear regression on each source’s visitation records separately. The coefficent describing the relationship between the cell data and the visitation counts ranged from .27-.32.

Supplementary Table S4: Linear regression results on each observational count source

|  | | | |
| --- | --- | --- | --- |
|  |  | | |
|  | Three Bays | Town | Narragansett |
| Cell data | 0.294^**^ | 0.323^***^ | 0.274^***^ |
|  | (0.112) | (0.035) | (0.012) |
|  |  |  |  |
| Temperature (°F) | -19.603 | 5.984^*^ | 38.806^***^ |
|  | (51.799) | (3.394) | (8.055) |
|  |  |  |  |
| Precipitation (inches) | -1798.98 | -33.86 | -144.03 |
|  | (3,913.899) | (57.685) | (105.958) |
|  |  |  |  |
| Area (m^2^) | 0.003 | -0.0001 |  |
|  | (0.002) | (0.0003) |  |
|  |  |  |  |
| Constant | 2864.21 | 33.953 | -2,998.80*** |
|  | (7,004.618) | (280.849) | (569.853) |
|  | | | |
| Observations | 72 | 234 | 86 |
| R^2^ | 0.361 | 0.516 | 0.957 |
| Residual Std. Error | 118.069 (df = 63) | 249.188 (df = 220) | 314.187 (df = 73) |
| F Statistic | 4.443^***^  (df = 8; 63) | 18.006^***^  (df = 13; 220) | 136.835^***^  (df = 12; 73) |
|  | | | |
|  | ^*^p^**^p^***^p<0.01 | | |

*Note:* Each column contains observations from each source of visitation observation information. Dummy variables are included in all regressions for month and day of the week. R-squared= % of in-sample variance explained.

REFERENCES

1. United States Environmental Protection Agency BEACON. <https://www.epa.gov/waterdata/beacon-20-beach-advisory-and-closing-online-notification> (2018).
