# Supplementary material for: Using data derived from cellular phone locations to estimate visitation to natural areas: An application to water recreation in New England, USA
Source: PLoS One. 2020 Apr 30;15(4):e0231863. doi: 10.1371/journal.pone.0231863 (PMC7192446; doi:10.1371/journal.pone.0231863)
Supplement: Attachment — Submitted filename: Response to Reviewers.docx [file pone.0231863.s003.docx]

**Response to Reviewers**

**Reviewer 1:**

Reviewer #1: I really appreciate the efforts to address my comments for the original submission. The quality of this manuscript is significantly improved. I particularly like how you acknowledge and discuss the limitation of the data/method and how people should use caution when using cell data to address real-world problems.
Some trivial suggestions:

1) Can you name different types of data in term of data collection method, instead of "small", "medium", and "large"?

We considered this, but the current way was the most clear and concise way to refer to the different groups of coastal sites. The scale of the site and visitation we believe to also be an important distinguishing feature, especially in future use of the data. The size also corresponds to the counting method for practical observational reasons which we discuss in depth now starting on line 193.

2) The ethics statement breaks the transition between methods and results. It should be moved to the end of the manuscript.

It is an odd place for it, but when submitting our revisions, we were instructed by the editor to put it there:

“Your ethics statement must appear in the Methods section of your manuscript. If your ethics statement is written in any section besides the Methods, please move it to the Methods section and delete it from any other section.”

We will ask if we can put it at the end of the manuscript when we submit this version, but it might be the journal’s format to have it in the methods.

**Reviewer 2**

The authors addressed most of my comments in the revision, and the manuscript has been greatly improved. I only have one minor comment- I suggest adding a thorough discussion on the data quality issues (e.g., precision, accuracy, biases) that occurred in the study, as well as the uncertainty caused by modeling fitting. You can also clarify the collinearity and spatial autocorrelation issues in the main text and point the readers to the supplementary materials for more details.

In addition to the discussion of the accuracy issues in the cell data, we added a longer discussion in the results section about the compounding sources of bias and inaccuracies starting on line 359:

“We used the most accurate and unbiased of the candidate statistical models for prediction. However, there are several sources of potential inaccuracies and biases in estimating visitation in this way that are not incorporated in the metrics of model goodness-of-fit. The observational visitation counts contain their own uncertainties and potential biases based on their sampling design and counting methods. By calibrating and validating to those counts, we may be carrying over those issues to our estimates of visitation. In addition, the cellular data contains uncertainties resulting from the geospatial accuracy of the device locations, our geographic definition of the sites, and the methods of expansion from the device sample to population-level estimates. More applications using cell datasets are needed to understand these limitations combined with additional and more consistent collections of visitation observations for calibration.

The models we fit may also be susceptible to spatial autocorrelation issues resulting from the cell dataset if there are variations in how the data represents visitation geographically. Spatial autocorrelation in models potentially inflates goodness-of-fit estimates, can bias parameters, and reduces predictive performance. We have controls in the models for each group of sites, which are geographically clustered to alleviate some of the potential issue. Similarly, collinearity in the covariates could potentially cause poor predictions, attributing predictive information to the wrong covariate, for example. We checked for this issue in a few ways by building up the model covariates sequentially starting with cell data alone and adding covariates. This led to little change in the relationships between observations and cell data counts (see Supporting Information for more model details and variations). We also consistently found good out-of-sample goodness-of-fit metrics in a cross validation, giving us more confidence that spatial autocorrelation and collinearity were likely not an issue in the models’ predictive performance.”

We also have a discussion point, pointing out the need for more and more consistent, unbiased collection of observational visitation data in traditional means. The lack of which holds back the new methods, like using cell datasets. Starting on line 424:

“While cell data-derived information is an exciting development for researchers and managers, counterintuitively, we found attempting to use it for a practical application only further motivated the need to take more accurate, consistent and unbiased observations of visitation using traditional methods. Modeling methods are hindered by the lack of availability of training datasets and would be greatly improved by larger and more uniformly collected observations. This is especially true regarding machine-learning algorithms [47]. For example, with small and practical tweaks in the way visitation records are collected at water-access areas, such as collecting periodic counts of cars and people at specific times, visitation records could become more harmonized and useful [12,41]. From there, visitation proxies like cell data or social media-based models can provide a platform for spatial and temporal extrapolation across broad geographies, as we demonstrate here. The need for such models is not confined to water-access visitation, as it is relevant to many other similar policy contexts, for example, at national parks or urban green spaces. The differences in how well our models fit depending on the visitation data source, with larger more popular locations fit better than small, should be cautionary to approaches applying the cell data-derived visitation estimates to settings where there is no similar observational data for comparison.”
